# Menthol- and thymol-based ciprofloxacin derivatives against *Mycobacterium tuberculosis*: in vitro activity, lipophilicity, and computational studies

**DOI:** 10.1038/s41598-023-43708-4

**Published:** 2023-09-28

**Authors:** Daniel Szulczyk, Mateusz Woziński, Michał Koliński, Sebastian Kmiecik, Agnieszka Głogowska, Ewa Augustynowicz-Kopeć, Michał A. Dobrowolski, Piotr Roszkowski, Marta Struga, Krzesimir Ciura

**Affiliations:** 1 Chair and Department of Biochemistry, The Medical University of Warsaw, 02-097 Warsaw, Poland; 2 Department of Physical Chemistry, Medical University of Gdańsk, 80-416 Gdańsk, Poland; 3 Bioinformatics Laboratory, Mossakowski Medical Research Institute, Polish Academy of Sciences, 5 Pawinskiego St., 02-106 Warsaw, Poland; 4 Faculty of Chemistry, Biological and Chemical Research Centre, University of Warsaw, 02-089 Warsaw, Poland; 5 Department of Microbiology, National Tuberculosis and Lung Diseases Research Institute, 01-138 Warsaw, Poland; 6 Faculty of Chemistry, University of Warsaw, Pasteura 1, 02-093 Warsaw, Poland; 7 QSAR Lab Ltd., Trzy Lipy 3 St., 80-172 Gdańsk, Poland

**Keywords:** Drug discovery and development, Bacterial infection

## Abstract

In this work, we investigated the antitubercular properties of Ciprofloxacin derivatives conjugated with menthol and thymol moieties. For the sixteen derivatives, we established minimal inhibitory concentrations (MIC) using isolates of *Mycobacterium tuberculosis* that were resistant or susceptible to other antibiotics. For the most potent compound 1‐cyclopropyl‐6‐fluoro‐7‐{4‐[6‐((1R,2S,5R)‐2‐isopropyl‐5‐methylcyclohexyloxy)‐6‐oxohexyl]piperazin‐1‐yl}‐4‐oxo‐1,4‐dihydroquinoline‐3‐carboxylic acid (**6**), we determined fractional inhibitory concentration index (FICI) values to confirm antibacterial susceptibility and synergistic effects with other reference drugs. In addition, chromatographic studies of all the derivatives demonstrated a significant three to four-fold increase in lipophilicity and affinity to phospholipids compared to Ciprofloxacin. Finally, we conducted structure-based studies of the investigated compounds using molecular docking and taking into account protein target mutations associated with fluoroquinolone resistance. In summary, our findings indicate that the investigated compounds possess tuberculostatic properties, with some showing similar or even better activity against resistant strains compared to reference drugs. Increased lipophilicity and affinity to phospholipids of the new derivatives can offer several advantages for new drug candidates, beyond just improved cell membrane penetration. However, further studies are needed to fully understand their safety, efficacy, and mechanism of action.

## Introduction

Tuberculosis (TB), caused by *Mycobacterium tuberculosis* remains a burning health issue, still being the most lethal infectious disease. To show the scale of TB epidemiology, an estimated 6.4 million people developed active TB^1^, of which 1.6 million died in 2021 compared to the 3.7 million deaths caused by COVID-19 in the same year^2^. Moreover, we need to remember that some high-risk populations can be more affected such as human immunodeficiency virus (HIV) patients. Only in 2017 were more 300,000 HIV-positive deaths reported^3^. It needs to be pointed out that part of the human population is infected with latent TB infection (LTBI), asymptomatic and nontransmissible, which can remain inactive but not in all individuals.

Treatment of TB is usually managed for a total of 6 months through directly observed therapy (DOT), where the two first months must contain four medicines (isoniazid, rifampicin, pyrazinamide, and ethambutol) and the next 4 months the use of two (isoniazid, rifampicin). This regimen is reserved for drug-susceptible tuberculosis. Unfortunately, TB resistance occurs and can be classified into five categories: Isoniazid-resistant TB, Rifampicin-resistant (RR-TB), multidrug‐resistant TB (MDR‐TB), pre-extensively drug‐resistant TB (pre-XDR‐TB) and extensively drug‐resistant TB (XDR-TB)^4^. Considering the epidemiology scale and TB resistance to commonly used medicines, there is a constant need for the development of new antitubercular agents. In the last years, new treatment options were approved by regulatory agencies (FDA and/or EMA) such as: Bedaquiline, Delamanid, and Pretomanid.

**Figure 1:**
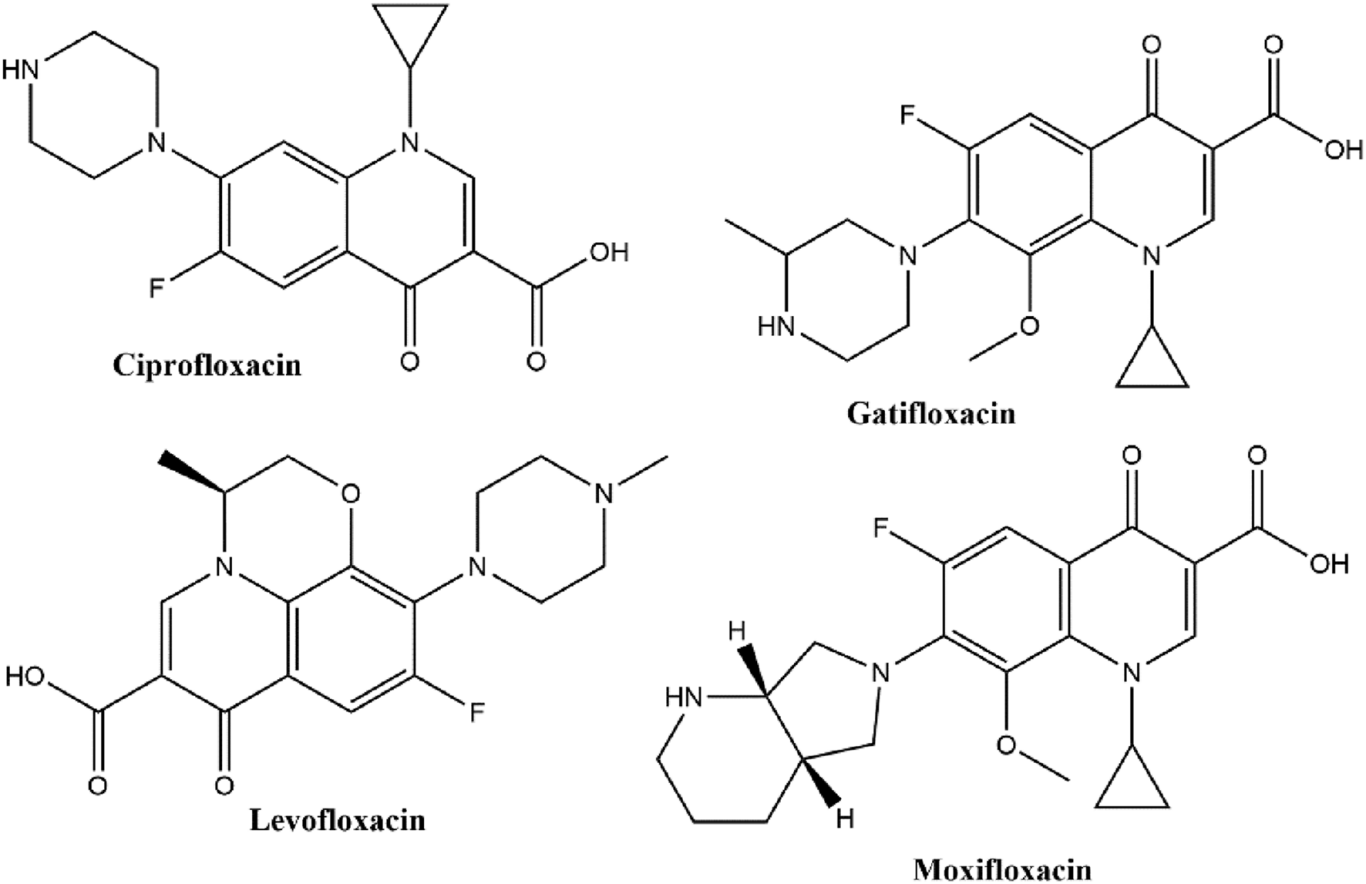
Structures of antibacterial fluoroquinolones.

Additionally, some candidates are currently in clinical trials. Another approach is to study or to use off-label already marketed antibiotics. Some of the compounds showed promising results: Clofazimine, Levofloxacin, Linezolid, Nitazoxanide, Moxifloxacin, Ciprofloxacin, Rifapentine, Gatifloxacin, and Rifampicin. Four of those mentioned are fluoroquinolones (Fig. 1), a class of very potent, broad‐spectrum synthetic antimicrobial agents that are currently being explored for the treatment of TB^5,6^. Most advanced in development are Moxifloxacin and Gatifloxacin, representing the fourth generation of quinolones antibiotics. All fluoroquinolones generally have a similar mechanism of action that targets DNA gyrase in Gram‐negative bacteria and topoisomerase IV in Gram‐positive bacteria^7^. There are used in clinical practice as an adjuvant drugs rather than in monotherapy or as second‐line drugs for the treatment of drug-resistant TB.

There are five TB-related resistance mechanisms: DNA gyrase mutations, drug efflux pumps, bacterial cell wall thickness, and pentapeptide proteins (MfpA)-mediated gyrase regulation in *M. tuberculosis*^8^. Increased antitubercular potential might be achieved due to enhanced lipophilicity of synthesized Ciprofloxacin conjugates. The drug molecule needs to penetrate multiple membranes present in the intracellular bacteria to reach the fluoroquinolone target, gyrase, or topoisomerase IV. Thus, the strong antimycobacterial effect can be achieved as a result of hydrophobic capacity which facilitates the transport of the drug across membranes^9^. We believe that synthesized dual-action Ciprofloxacin conjugates can possess increased efficacy towards bacterial strain, lower cytotoxicity, and in parallel, limited bacteria resistance.

The lipophilicity of medicinal substances is measured by their distribution behavior in a biphasic system, either a liquid–liquid or solid–liquid system. Currently, indirect chromatographic methods are mainly applied since they showed significant advantages compared to classical partition measurement between water and *n*-octanol via the directed shake-flask method. The main benefits of the chromatographic approach are short analysis time, full automatization of the analysis process, and low consumption of organic solvent. Consequently, chromatographic methods, especially reserved phase high-performance liquid chromatography (RP-HPLC), dominated lipophilicity determinations both in the academic and pharmaceutical industry environment.

Considering that fluoroquinolones (FQs) antibiotics are promising starting structures in the anti-TB drug discovery pipeline, our team has decided to continue the broad study of Ciprofloxacin derivatives and investigate the anti-TB properties of previously synthesized menthol and thymol hybrids^10^. Biological assays covered reference strain of *M. tuberculosis* (H37Rv) and also clinical isolates; Linezolid susceptible SIR 168/22 and Linezolid resistant SIRE 22/22 collected from patients of the National Tuberculosis and Lung Diseases Research Institute. Additionally, we studied the interaction between the most potent derivative and reference drugs used in the treatment of TB. Both, menthol and thymol affect permeability and depolarize the cytoplasmic membrane, thus it can be assumed that its conjugates should show membrane-related antibacterial activity. Therefore, in this study, we investigated their lipophilicity and affinity to phospholipids using well-established RP-HPLC methods. To better understand the effects of molecular properties on experimentally determined lipophilicity and affinity to phospholipids we applied the quantitative structure-retention relationship (QSRR) approach. Moreover, molecular docking experiments were performed to assess the typical enzymatic target of action related to the Ciprofloxacin part of conjugates.

## Results

### Synthesis

The mechanism underlying the antimicrobial effects of phenolic oils, which include monoterpenes, has been thoroughly investigated and confirmed. In broad terms, the primary mode of action is attributed to their capacity to disrupt bacterial biomembranes, leading to structural damage (a toxic impact on membrane integrity). Nevertheless, it's worth noting that multiple interactions with bacterial cells contribute to their antimicrobial activity. In previous^10^ and current studies, menthol and thymol moieties were chosen for several interactions with bacterial cells, such as: damage to membrane proteins, reduced ATP synthesis, increased membrane permeability and membrane fluidity causing leakage of ions, a decrease in the pH gradient across the cytoplasmic membrane^11,12^.

Initial molecular docking studies, an established pharmacophore model, and preliminary observations from X-Ray studies revealed that a common fluoroquinolone structure is decisive (see Section "X-ray data") in designing new antimicrobials based on that skeleton. Substituents present in structures of Moxifloxacin and Levofloxacin are limiting chemical modifications. For this study, we have picked Ciprofloxacin over Gatifloxacin as starting material, although both possess accessible piperazinyl moiety. Our previous examination indicated that thymol and menthol Ciprofloxacin derivatives showed promising antibacterial activities and low toxicity to eukaryotic cells^10^. Encouraged by the results, we have decided to continue our study.

### In vitro tuberculostatic activity

Sixteen derivatives of Ciprofloxacin were transferred to estimate antitubercular properties. Primarily, minimal inhibitory concentrations (MIC) were established, using the *M. tuberculosis* H_37_Rv strain (ATCC 25618) and two “wild” strains isolated from tuberculosis patients: one (168/22) resistant to Streptomycin, Isoniazid, and Rifampicin (SIR) and susceptible to Ciprofloxacin and the another (22/22) resistant to Streptomycin, Isoniazid, Rifampicin, and Ethambutol (SIRE) as well as Linezolid, Ciprofloxacin, Moxifloxacin, and Levofloxacin. Results for both, menthol and thymol derivatives are gathered in Table 1.

**Table 1:** The activity of Ciprofloxacin derivatives and reference drugs against *Mycobacterium tuberculosis* strains (standard, susceptible, resistant), expressed by minimal inhibitory concentrations (μg/mL).

Compound	Minimal inhibitory concentration (μg/mL)		
	*M. tuberculosis* H_37_Rv	*M. tuberculosis* SIR Ciprofloxacin susceptible 168/22	*M. tuberculosis* SIRE Ciprofloxacin resistant 22/22
**1**	32	256	32
**2**	0.5	2	16
**3**	2	2	16
**4**	0.5	0.5	16
**5**	4	4	16
**6**	1	1	8
**7**	256	256	256
**8**	4	4	256
**9**	256	256	128
**10**	1	1	128
**11**	1	1	64
**12**	0.5	0.5	32
**13**	2	2	32
**14**	0.5	0.5	16
**15**	64	64	256
**16**	4	4	256
Ciprofloxacin	0.5	0.0625	16
Isoniazid	0.0625	0.5	32
Linezolid	0.003	0.007	8
Rifampicin	0.5	64	256
Streptomycin	0.25	64	32
Ethambutol	0.25	0.5	4

*SIR* resistant to streptomycin, isoniazid, and rifampicin, *SIRE* resistant to Streptomycin, Isoniazid, Rifampicin, and Ethambutol.

Among investigated, only four derivatives **1**, **7**, **9**, and **15** should be considered as not active toward standard and susceptible strains. Most promising results were observed for derivatives **4**, **6**, **12,** and **14** and consequently for all used strain. The rest of the compounds can be described as possessing moderate activity. Six reference antibiotics were also evaluated including Ciprofloxacin. What is interesting, all of them were highly potent against standard strain. Then, Rifampicin and Streptomycin showed poor activity in the case of susceptible bacteria. Finally, when investigated towards resistant strain, all of the reference drugs showed very decreased activity. Rifampicin result confirms no activity against SIRE 22/22 isolate. In general, investigated compounds showed similar or higher levels of activity as reference drugs. The best MIC result (8 μg/mL) against resistant clinical isolate was observed for derivative **6**, while Ciprofloxacin MIC was 16 μg/mL. Linezolid showed the same level of action. Structural trends or preferences were not observed except for one. Derivatives containing linkers -oxobutyl and -oxohexyl were rather more active than compounds possessing short linkers such as -acetyl. What should be underlined, is that all derivatives were not cytotoxic in MTT assay. As an example, the selectivity index (3.4) was determined for compound **6**, with IC_50_ 29.5 ± 2.1 µM against human colon cancer cells (HCT-116) and with no cytotoxic effect on human immortal keratinocyte cell line from adult human skin (HaCaT)^10^.

### Synergism—antagonism evaluation

Derivative **6** was transferred to an additional study, based on the best result towards *Mycobacterium tuberculosis* strain resistant to Ciprofloxacin*.* This compound reached the level of activity of reference Linezolid and was twofold more potent than Ciprofloxacin (MIC 8 and 16 μg/mL, respectively), therefore among other derivatives was most suitable for that test. The results of the tests are presented in Table 2.

**Table 2:** Results of synergism/antagonism study for most potent compound **6** combined with reference drugs.

MIC (μg/ml) for derivative 6 in the presence of reference drug		MIC (μg/ml) for reference drug in the presence of derivative 6		FICI values
*M. tuberculosis* H_37_Rv				
** 6**/Ciprofloxacin	1	Ciprofloxacin/**6**	0.25	1.5
** 6**/Isoniazid	0.5	Isoniazid/**6**	0.0625	1.5
** 6**/Linezolid	0.5	Linezolid/**6**	0.0015	1.0
*M. tuberculosis* SIRE Ciprofloxacin resistant 22/22				
** 6**/Ciprofloxacin	4	Ciprofloxacin/**6**	8	1.0
** 6**/Isoniazid	8	Isoniazid/**6**	32	2.0
** 6**/Linezolid	4	Linezolid/**6**	4	1.0
*M. tuberculosis* SIR Ciprofloxacin susceptible 168/22				
** 6**/Ciprofloxacin	0.5	Ciprofloxacin/**6**	0.031	0.996
** 6**/Isoniazid	0.5	Isoniazid/**6**	0.5	1.5
** 6**/Linezolid	0.5	Linezolid/**6**	0.0035	1.0

The obtained FICI values were used to determine whether synergism (FICI ≤ 0.5), indifference (> 0.5 FICI ≤ 4), or antagonism (FICI > 4) occurred between the tested agents.

We found the results of the evaluation interesting since indifference was observed in all cases. The FICI values for compound **6** and drug combinations were in the range of 0.996 to 2.0 for all strains (Table 2). Combinations of derivative **6** with Ciprofloxacin or Linezolid resulted in a twofold decreased MIC of the antibiotics. The MIC value obtained for Ciprofloxacin decreased from 0.5 to 0.25 μg/mL on *M. tuberculosis* H_37_Rv, from 16 to 8 μg/mL on *M. tuberculosis* Ciprofloxacin resistant strain (22/22), and from 0.062 to 0.031 μg/mL on *M. tuberculosis* Ciprofloxacin susceptible strain (168/22). The FICI values for the pair of derivative **6** and Ciprofloxacin were in the range of 0.996 to 1.5. Similarly, the MIC for Linezolid decreased from 0.003 to 0.0015 μg/mL on *M. tuberculosis* H_37_Rv, 8 to 4 μg/mL on *M. tuberculosis* Ciprofloxacin resistant strain (22/22), and from 0.007 to 0.0035 μg/mL on *M. tuberculosis* Ciprofloxacin susceptible (168/22). The FICI values in the case of all strains were 1.0. For all strains tested, the combination of compound **6** with isoniazid did not change their MIC INH values and the FICI values were between 1.5 and 2.0.

### Lipophilicity and affinity to phospholipids

Numerous studies have suggested that utilizing chromatography is an effective method for evaluating the lipophilicity of FQs antibiotics and their derivatives. Various investigations have employed different techniques, including thin-layer chromatography, RP-HPLC, and even the use of an immobilized artificial membrane (IAM) column. Drawing from our prior expertise in estimating the lipophilicity of FQs derivatives, we have opted for a protocol centered around the chromatographic hydrophobicity indices (CHI) as introduced by Valko and his colleagues^13–19^.

The received CHI log D and log *k*_IAM_ were collected in Table 3. The results indicated that investigated Ciprofloxacin derivatives have significantly higher lipophilicity and affinity to phospholipids than parent Ciprofloxacin. In this context, CHI values obtained on C_18_ columns are off-scale values, but it should be stressed that this method allows for extrapolation. The calculated logD value was much lower for the majority of compounds, indicating that they are promising drug candidates. The exception was molecules **5**, **8**, **10**, and **12**, in which the calculated logD ranges from 8.33 to 10.05. Although high lipophilicity may limit the medical use of the analyzed compounds, it is worth noting that the increase in affinity for phospholipids was not as significant as lipophilicity. In the case of CHI_IAM_, the scale is from 0 to 50. The analyzed compounds are in the upper part of the scale or slightly above it. These values suggested that obtained Ciprofloxacin derivatives can strongly interact with biological membranes, which may contribute to the opening of a new, biological membrane-oriented mechanism of action.

**Table 3:** Combined results from biomimetic chromatography study and calculated lipophilicity indices of Ciprofloxacin derivatives using the chemicalize software.

No	CHI_IAM_	log *k*_IAM_	CHI_C18_	CHI logD	logD pH 7.4**
**1**	45.54	2.47	112.24	4.43	2.49
**2**	46.88	2.53	127.44	5.22	2.25
**3**	49.56	2.65	129.99	5.36	2.69
**4**	53.32	2.82	132.45	5.49	2.93
**5**	48.95	2.62	134.85	5.61	8.51
**6**	51.47	2.74	139.48	5.86	3.02
**7**	55.54	2.92	170.54	7.49	3.26
**8**	57.85	3.02	179.66	7.97	8.33
**9**	43.58	2.38	129.41	5.33	3.47
**10**	42.88	2.35	111.26	4.37	10.05
**11**	44.44	2.42	113.95	4.52	3.71
**12**	45.75	2.48	115.60	4.60	9.68
**13**	46.96	2.53	117.28	4.69	2.41
**14**	50.29	2.68	121.48	4.91	2.97
**15**	45.54	2.47	135.98	5.67	3.20
**16**	49.33	2.64	144.84	6.14	2.67
Ciprofloxacin*	19.70	1.31	35.61	0.40	-0.88

*Determined in an earlier study^15^.

**Calculated by chemicalize software.

### Quantitative structure-retention relationship

To find molecular properties of Ciprofloxacin derivatives that influence the affinity to phospholipids and lipophilicity, QSRR models were calculated applying MLR regression mode. MLR offers significant advantages, like direct relation to original data and straightforward interpretation. Descriptors selection was supported by GA, which is an optimization method inspirited by natural selection. Briefly, where GA is linked with regression methods, the population of variables is initially created randomly. The next step calculates the model performance and survives the model with the highest metrics. Loop for further crossover and mutation specified the properties of the algorithm. The best-obtained models are listed below:

$$ {\text{log}}k_{{{\text{IAM}}}} = {2}.{593} + 0.{\text{156 Charge pH}}_{{{7}.{4}}} - 0.{\text{114 Aromatic ring count}} $$

$$ {\text{R}}^{{2}} = 0.{855},{\text{ Q}}^{{2}}_{{{\text{LOO}}}} = 0.{742},{\text{ Q}}^{{2}} = 0.{723},{\text{ RMSE}}_{{{\text{CV}}}} = 0.0{72},{\text{ RMSEp}} = 0.0{9}0 $$

$$ {\text{CHI log D}} = {5}.{38} + 0.{\text{915 Maximum projection area}} - 0.{\text{422 Aromatic ring count}} $$

$$ {\text{R}}^{{2}} = 0.{9}0{3},{\text{ Q}}^{{2}}_{{{\text{LOO}}}} = 0.{825},{\text{ Q}}^{{2}} = 0.{745},{\text{ RMSE}}_{{{\text{CV}}}} = 0.{399},{\text{ RMSEp}} = 0.{536} $$

The high values of R, Q^2^, Q^2^_LOO_, and small RMSE_CV_ and RMSE_P_ confirmed that obtained models are not well-fitted to the training data, but also showed prediction power. Moreover, the established models were subjected to a y-randomization test to confirm their robustness (Fig. S1). The applicability domain of the obtained models is presented as William’s plot (Fig. S1). Summarizing all statistical figures, the y-randomization test and the presence of all points in the model domain indicate that the obtained models meet the criteria.

The character of molecular descriptors allows for mechanistic interpretation of obtained models. Generally, the count of the aromatic ring plays a pivotal role in the case of both lipophilicity and affinity to phospholipids. Maximum projection area influences lipophilicity determined chromatographically, which also has a great sense since the greater the projection area available for interactions with the hydrocarbon chains, the more strongly the molecule is bound to the mobile phase. Compared to the neutral character of the C_18_ bonded stationary phase, IAM stationary phase has a zwitterionic character. The positively charged choline moieties are located in the outer part of the phosphatidylcholine layer, and negatively charged phospholipids are in the inner part. This explains why the charge of molecules determines the interaction between IAM and target Ciprofloxacin derivatives.

### Molecular docking

In the docking study, we aimed to characterize the binding modes of the Ciprofloxacin derivatives together with the effect of DNA gyrase mutations associated with drug resistance. Three models of DNA gyrase were constructed (Table 4), including a combination of eight-point mutations associated with drug resistance (what needs to be underlined, the introduced mutations are among the most significant described in the literature, but due to the multitude they are exemplary and do not exhaust all possibilities). The docking results for the three models of DNA gyrase (1) point mutations in the GYR A subunit, (2) point mutations in the GYR B subunit, (3) point mutations in GYR A and GYR B) were compared to the results of docking to wild-type DNA gyrase for a set of 14 ligands from previous studies. Derivatives **7**, **8**, **15**, and **16** were not included in the experiment due to structural substitution of the carboxylic group of base structure, which resulted in poor antimicrobial activity.

**Table 4:** Docking results for ciprofloxacin and its derivatives.

Compound number	Wild type^a^				Point mutations in the A subunit^b^				Point mutations in the B subunit^c^				Point mutations in the A and B subunits^b,c^			
	Number of members of the largest cluster	Binding energy [kcal/mol]	Energy range of the largest cluster [kcal/mol]	Ligand efficiency	Number of members of the largest cluster	Binding energy [kcal/mol]	Energy range of the largest cluster [kcal/mol]	Ligand efficiency	Number of members of the largest cluster	Binding energy [kcal/mol]	Energy range of the largest cluster [kcal/mol]	Ligand efficiency	Number of members of the largest cluster	Binding energy [kcal/mol]	Energy range of the largest cluster [kcal/mol]	Ligand efficiency
**1**	119	− 10.42	2.51	− 0.27	97	− 9.94	3.16	− 0.26	205	− 11.39	3.95	− 0.30	198	− 11.98	3.35	− 0.31
**2**	100	− 11.76	2.68	− 0.31	127	− 11.02	2.76	− 0.29	217	− 12.19	3.81	− 0.32	214	− 12.79	4.71	− 0.34
**3**	184	− 12.33	3.46	− 0.32	136	− 11.67	3.41	− 0.30	225	− 12.47	4.23	− 0.32	178	− 13.46	4.65	− 0.35
**4**	132	− 12.21	2.82	− 0.31	90	− 11.53	3.51	− 0.29	221	− 10.54	2.27	− 0.26	151	− 11.26	2.68	− 0.28
**5**	165	− 12.55	3.62	− 0.31	115	− 11.94	3.78	− 0.29	103	− 12.86	3.31	− 0.31	90	− 13.29	3.74	− 0.32
**6**	81	− 12.65	3.11	− 0.30	51	− 11.92	2.98	− 0.28	120	− 10.8	1.94	− 0.26	66	− 11.41	3.05	− 0.27
**9**	90	− 10.79	2.65	− 0.28	75	− 10.52	2.66	− 0.28	233	− 10.3	3	− 0.27	136	− 9.9	2.94	− 0.26
**10**	181	− 10.53	2.66	− 0.28	134	− 10.95	2.77	− 0.29	361	− 12.01	3.58	− 0.31	429	− 12.69	4	− 0.33
**11**	245	− 11.6	3.87	− 0.30	174	− 11.36	3.07	− 0.29	255	− 12.65	3.87	− 0.32	292	− 13.35	4.7	− 0.34
**12**	169	− 11.75	2.95	− 0.29	121	− 11.16	2.94	− 0.28	159	− 12.57	3.62	− 0.31	137	− 13.5	4.39	− 0.34
**13**	127	− 12.24	3.15	− 0.30	105	− 11.63	3.73	− 0.28	58	− 10.68	2.05	− 0.26	151	− 11.04	2.79	− 0.27
**14**	124	− 12.52	4.12	− 0.30	78	− 11.89	2.75	− 0.28	73	− 13.02	4	− 0.31	89	− 12.96	4.89	− 0.30
Ciprofloxacin	569	− 7.47	1.01	− 0.31	528	− 7.29	1.1	− 0.30	181	− 6.13	0.94	− 0.26	272	− 6.84	1.5	− 0.29

^a^Results from our earlier work^10^ for comparison.

^b^Point mutations in the A subunit (Gyr A) included: D89G, S90V, S91P, and D94A.

^c^Point mutations in the B subunit (Gyr B) included: R482G, N499G, T500H, and E501G.

**Figure 2:**
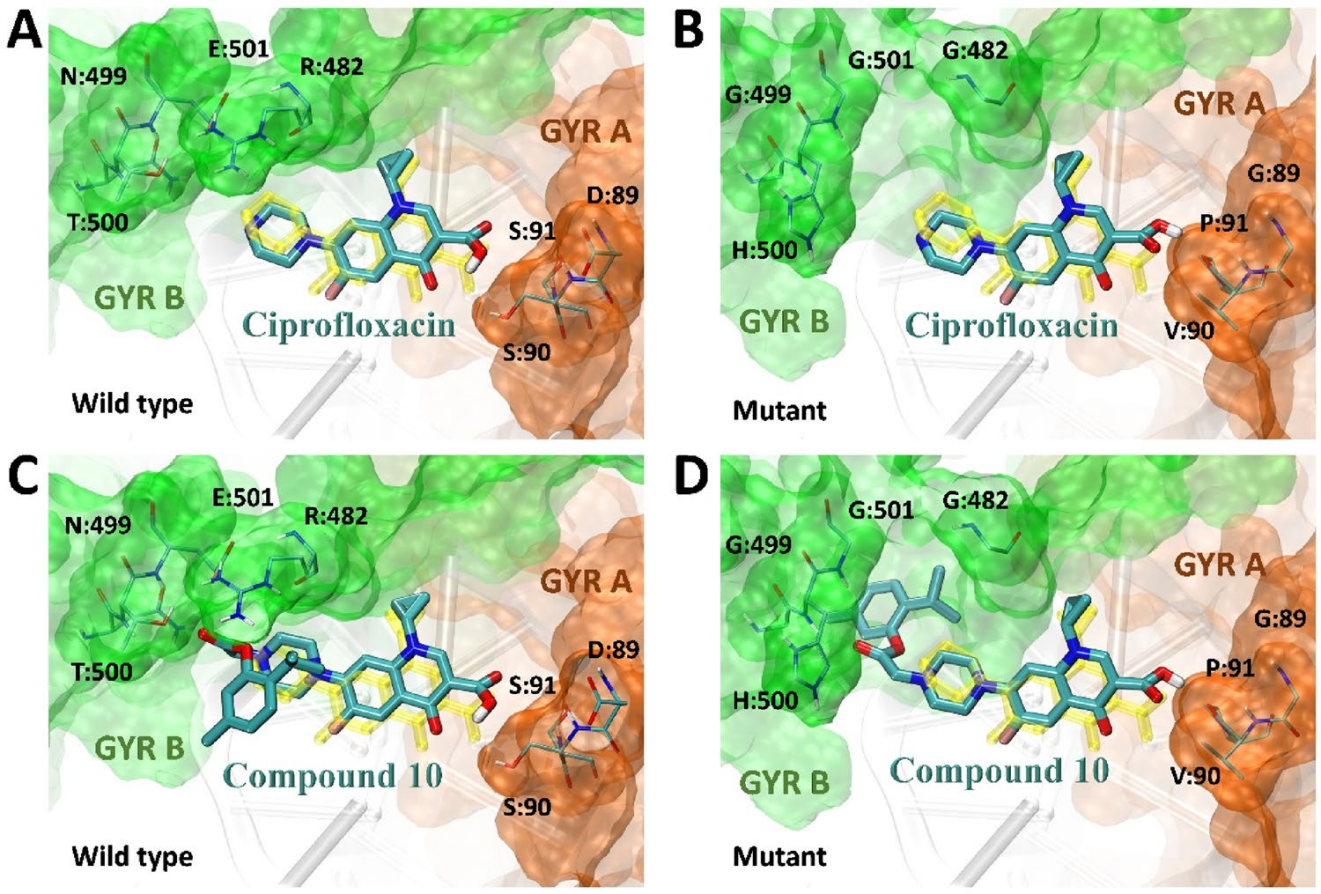
The binding modes were obtained from molecular docking for Ciprofloxacin (**A** and **B**) and compound **10** (**C** and **D**). Ligand binding modes are displayed for two different gyrase DNA models: one that presents the wild-type variant (left panels) and another that contains point mutations in both GYR A (D89G, S90V, S91P, and D94A) and GYR B (R482G, N499G, T500H, and E501G) subunits. The binding pocket is shown as an orange surface for GYR A and a green surface for GYR B. Docked ligands and surrounding amino acid chains are displayed in stick representation. For comparison, the yellow sticks depict the structure of Ciprofloxacin derived from the experimental structure (PDB ID: 5BTC^20^). Fragments of the DNA chain are displayed in gray cartoon representation.

The binding energy values and ligand efficiency were similar in all docked structures. The larger cluster size obtained for compound **10** docked to the DNA gyrase mutant may be a result of the increased size of the binding site in the region of GYR B, which includes the residues G:482, G:499, and G501 (see Fig. 2D).

**Figure 3:**
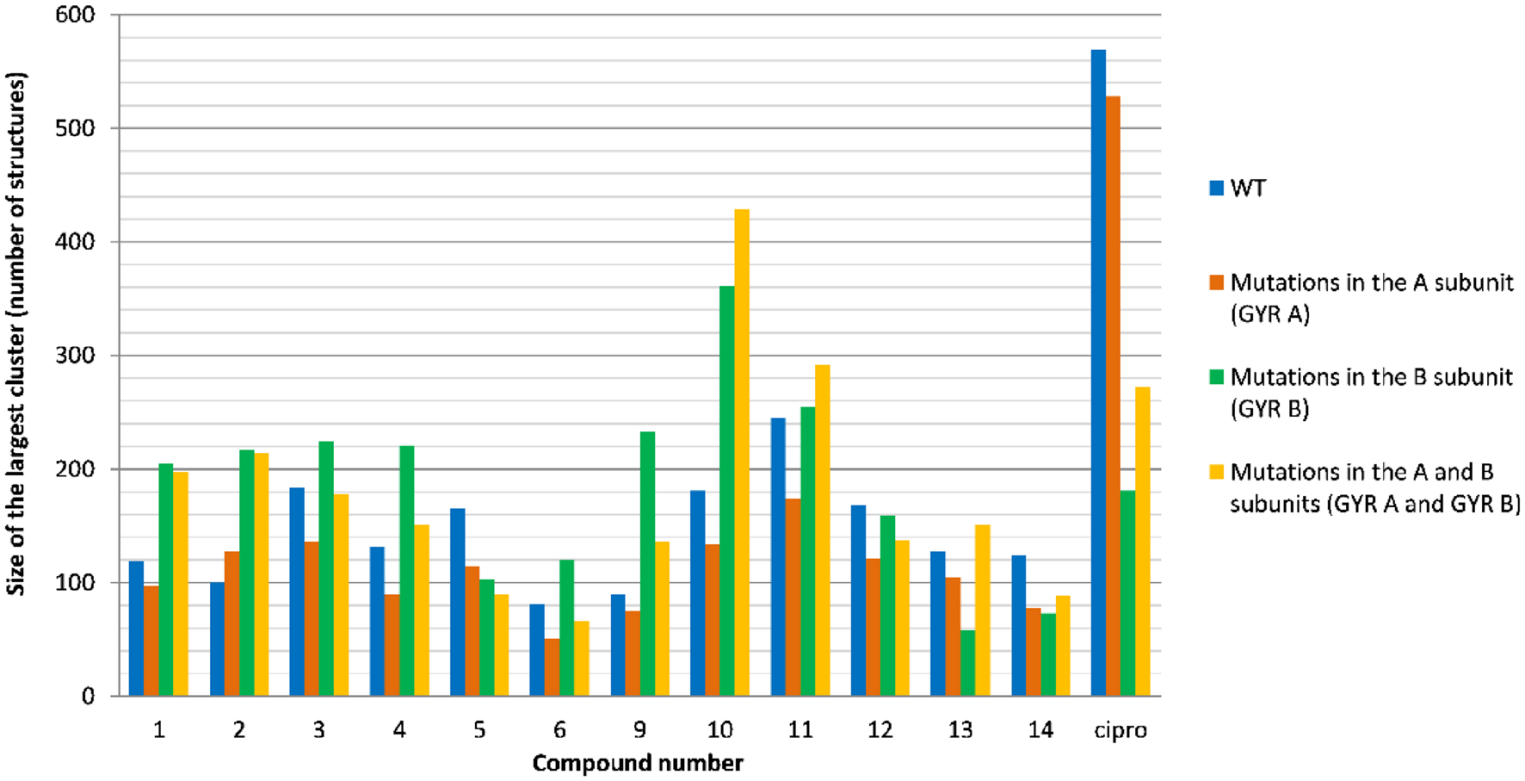
The largest clusters were obtained from docking simulations of Ciprofloxacin (Cipro) and its derivatives to four different gyrase DNA models. For each molecule four bars correspond to the largest cluster size resulting from docking to the following gyrase DNA model: (1) a model based on crystal structures PDB ID: 5BTC, which presents the wild-type form of DNA gyrase (blue bar); (2) a model with four-point mutations (D89G, S90V, S91P, and D94A) in the A subunit of DNA gyrase (orange bar); (3) a model with four-point mutations (R482G, N499G, T500H, and E501G) in the B subunit of DNA gyrase (green bar); and (4) a model that incorporates all of the described mutations in both the A and B subunits of DNA gyrase (yellow bar).

The point mutations significantly decreased the size of the largest clusters for Ciprofloxacin. On the other hand, the size of the largest clusters for compound **10** increased significantly (Fig. 3).

The underlying assumption is that the search algorithm of the docking software more easily identifies binding poses in wide energy wells compared to narrow wells. Therefore, the probability of identifying similar binding poses (reflected by the size of the largest cluster in Fig. 3) correlates with the width of the energy well and the accessible configurational entropy of the ligand in the binding site.

In summary, the docking results showing binding similar to the ciprofloxacin binding site suggest that the new compounds still target the DNA gyrase and that the observed resistance mechanism may be involved with mutations in the DNA gyrase.

### X-ray data

**Figure 4:**
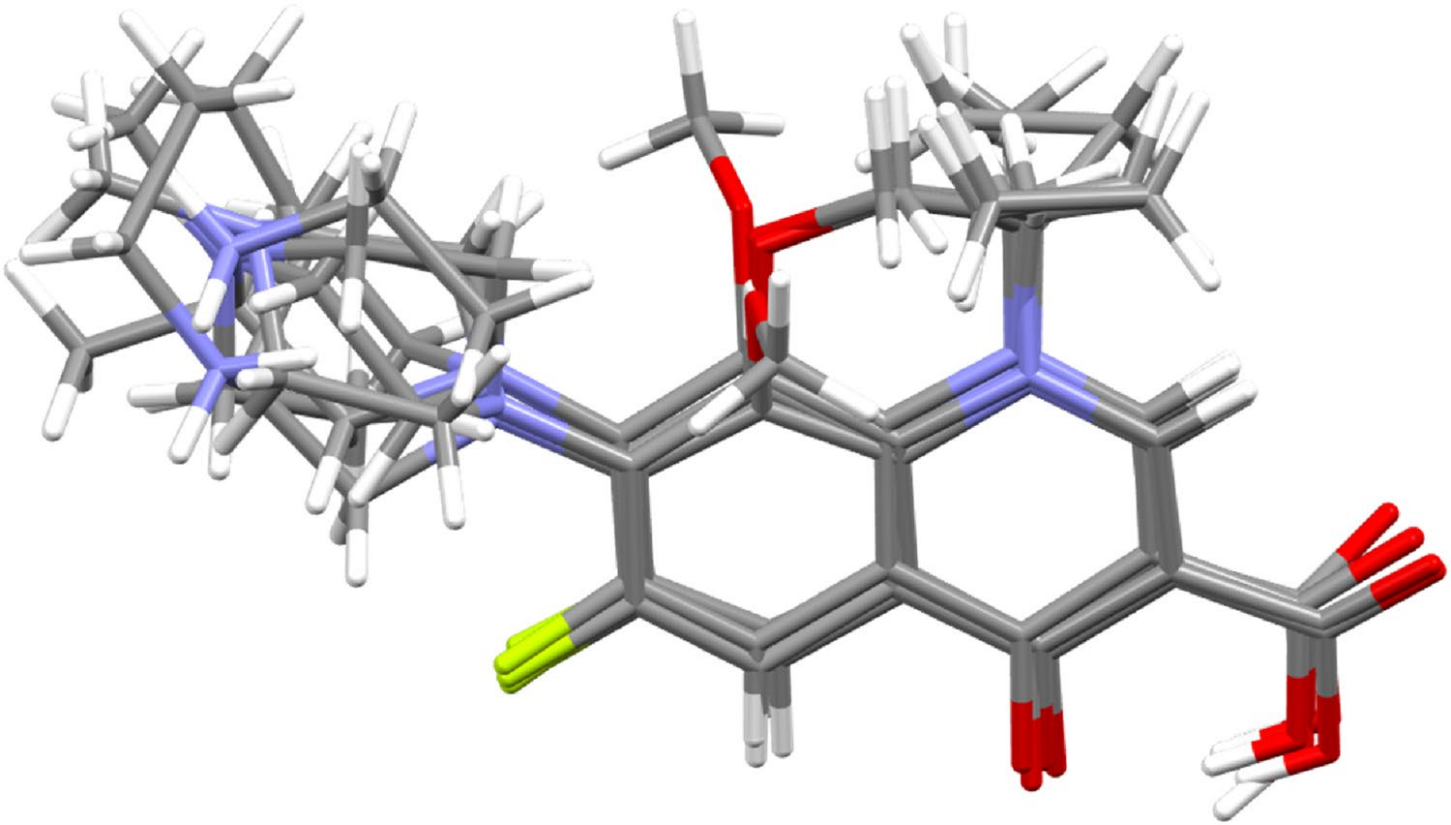
The superposition of Ciprofloxacin, Moxifloxacin, Gatifloxacin, and Levofloxacin (two polymorphs) molecules.

Five molecules of fluoroquinolone derivatives have been superimposed as can be seen in Fig. 4.

In all cases the core fluoroquinolone moiety is almost planar with angles between two best planes of adjacent rings varying from 2° to 7° and the molecules are stabilized by intramolecular hydrogen bond O–H…O from carboxyl to the keto group. Whereas, other substituents show high lability (e.g. cyclopropyl, methoxy, and piperazine).

**Figure 5:**
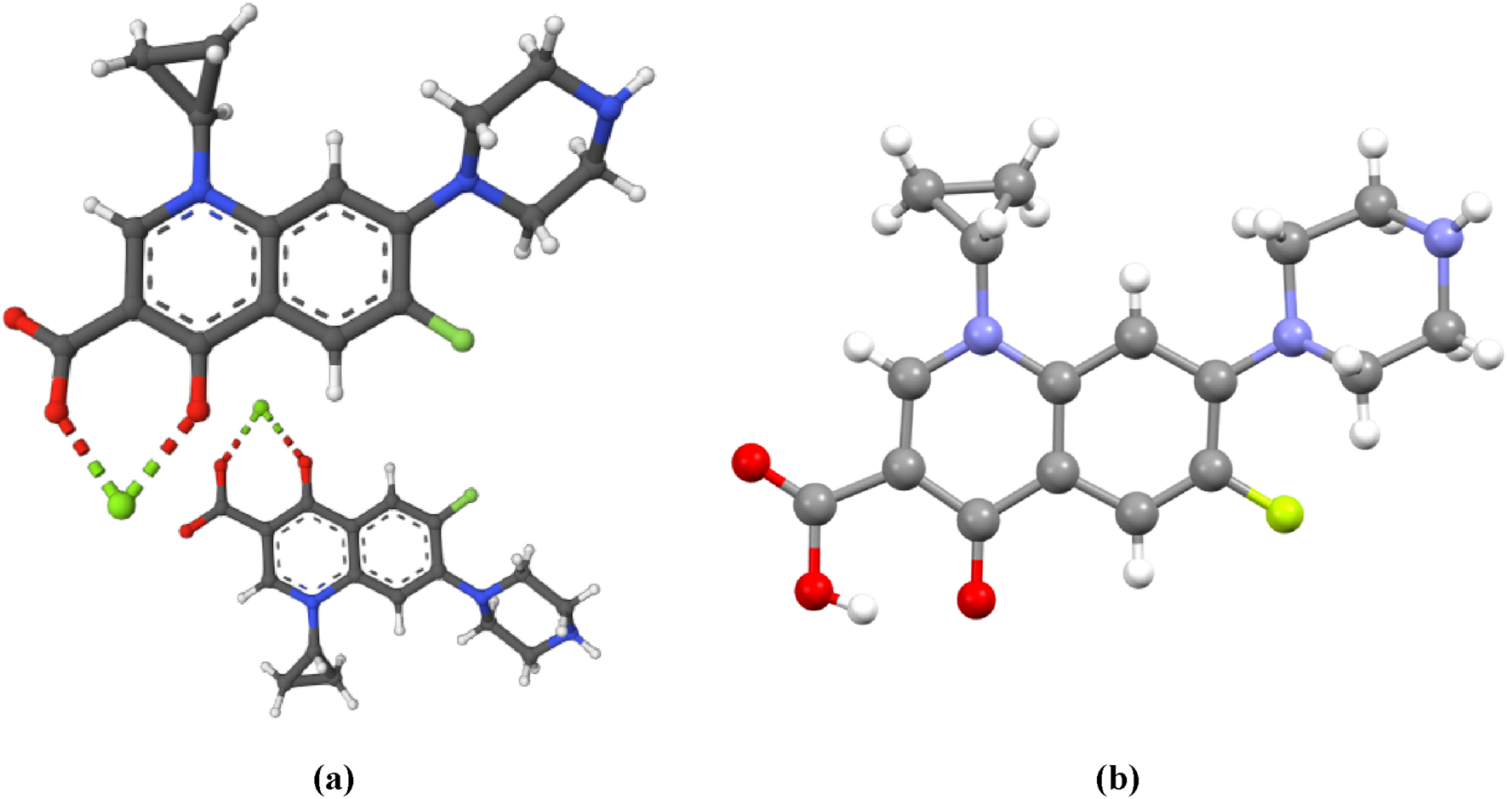
Ciprofloxacin molecule geometries in gyrase DNA complex (**a**) ^20^ and “pure” crystal (**b**) ^20^.

Solid state “pure” Ciprofloxacin geometry is similar to the one found in the gyrase DNA complex (Fig. 5) with obvious differences at cyclopropyl and piperazine substituents^20^. In this work, we attempted to crystallize the studied derivatives, unfortunately, no suitable single structures were obtained. What’s important, in the docking study, our compounds showed the same planar fluoroquinolone skeleton with mobile substituents (see Fig. 4) as in other studies mentioned above.

## Discussion

All the designed derivatives of Ciprofloxacin were assessed for their activity against Mycobacterium tuberculosis strains in a series of tests. Overall, the synthesized derivatives demonstrated activity levels comparable to the reference drugs. Compound **6** was included in a synergism-antagonism study, and the results of the Fractional Inhibitory Concentration Index (FICI) confirmed indifference. We observed that reference drugs lost efficacy against Ciprofloxacin-resistant SIRE 22/22 strain, while such a strong activity decrease was not observed for the greater part of derivatives. The best MIC result (8 μg/mL) against resistant clinical isolate was observed for derivative **6**, while Ciprofloxacin MIC was 16 μg/mL. Combinations of derivative 6 with Ciprofloxacin resulted in a twofold decreased MIC of the antibiotics. The reduction of MIC values may indicate beneficial interactions between derivative 6 and Ciprofloxacin, which in consequence may reduce side effects. The results obtained in this study may support the thesis of additional membrane interaction activity mechanism in parallel with bacterial DNA gyrase targeting.

A molecular docking experiment estimated the influence on Ciprofloxacin and its derivatives structures in case of changes in gyrase subunits amino acids sequences. Changes in gyrase subunits should be recognized as possible mutations producing acquired resistance to TB antibiotics. Intended point mutations significantly decreased the size of the largest clusters for Ciprofloxacin, while for derivative **10** the size of the largest cluster increased. Furthermore, binding energy values of Ciprofloxacin and investigated compounds do not differ significantly in the range of designed binding modes. Several reports indicated that the chromatographic approach can be considered an efficient tool for lipophilicity assessment of FQs antibiotics and their derivatives. Some reports are based on thin-layer chromatography^13,14^ while others applied RP-HPLC^15^ considering also immobilized artificial membrane (IAM) column^16^. Summarizing our previous experience in lipophilicity estimation of FQs derivatives^17,18^, we have chosen a protocol based on the chromatographic hydrophobicity indices (CHI) proposed by Valko and co-workers^19^. This method involves gradient elution using acetonitrile as an organic modifier of the mobile phase. It can be applied for typical C_18_ bonded silica gel, the gold standard for chromatographically determining lipophilicity, and more biologically relevant IAM columns. Moreover, since the experiments were conducted in physiological pH conditions, the established lipophilicity better reflects the lipophilic character of charged molecules, which are zwitterionic Ciprofloxacin derivatives.

Our chromatographic studies indicated that investigated Ciprofloxacin derivatives have significantly higher lipophilicity and affinity to phospholipids than parent Ciprofloxacin. High lipophilicity can be considered as both an advantage and a limitation. In the context of present in vitro results, increased lipophilicity and affinity to phospholipids might have the following effects: increased solubility in the MIC assays, improved cell membrane penetration, increased potency (by more efficiently interacting with the bacterial cell membrane and/or intracellular targets) or reduced efflux.

Based on the obtained QSRR models, we identify which molecular properties determine chromatographically measured lipophilicity and affinity to phospholipids. Generally, the count of the aromatic ring plays a pivotal role in the case of both lipophilicity and affinity to phospholipids. Maximum projection area influences lipophilicity determined chromatographically, which also has a great sense since the greater the projection area available for interactions with the hydrocarbon chains, the more strongly the molecule is bound to the mobile phase. Compared to the neutral character of the C_18_ bonded stationary phase, IAM stationary phase has a zwitterionic character. The positively charged choline moieties are located in the outer part of the phosphatidylcholine layer, and negatively charged phospholipids are in the inner part. This explains why the charge of molecules determines the interaction between IAM and target Ciprofloxacin derivatives.

On the other hand, there is no clear relationship between determined lipophilicity and biological activity. Derivatives **3** and **9** have practically the same CHI logD values but exhibit different biological activities, 2 versus 256 MIC, respectively. A similar situation occurs when we consider binding to phospholipids; we can also find pairs for active and inactive structures showing very similar log*k*_IAM_, for example, molecules **9** and **10** (2.38 vs. 3.35).

In conclusion, based on the information provided, it is difficult to make conclusions about the exact mechanisms of antituberculosis action of the new ciprofloxacin derivatives. However, the increase in lipophilicity and affinity to phospholipids may enhance the ability of the drug to penetrate cell membranes and reach its target site in *M. tuberculosis* cells. Also, more lipophilic compounds may also be less susceptible to efflux-mediated resistance. Future in vitro investigations performed on a broader group of fluoroquinolone derivatives should reveal more details on specific membrane interactions and confirm the role of additional mechanism of action. The presented results open new avenues in antituberculosis drug discovery and indicated new promising initial hybrids structures of a more lipophilic character.

## Materials and methods

### Synthesis

**Figure 6:**
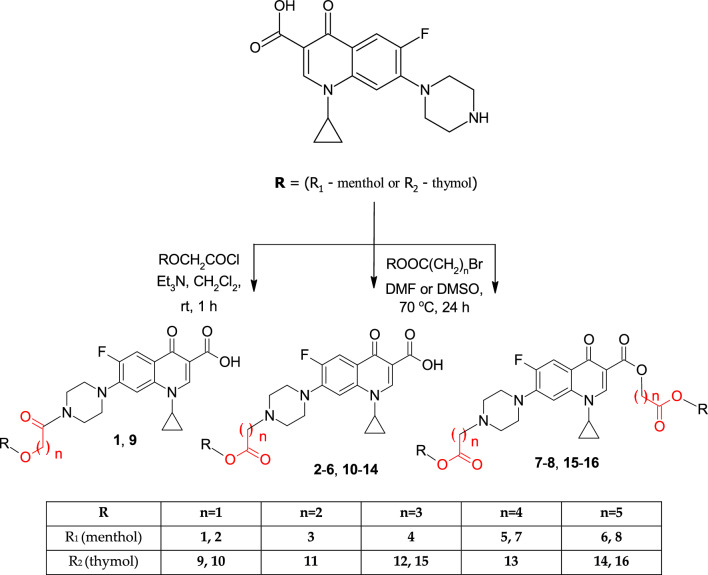
Structural modifications of ciprofloxacin.

The core structure of Ciprofloxacin was modified to improve antibacterial action. Three types of modifications were designed and synthesized (Fig. 6). Menthol and thymol moieties were attached to Ciprofloxacin with the usage of diverse carboxylic linkers. In general, derivatives were obtained via the condensation reaction of Ciprofloxacin and corresponding menthol or thymol esters. All details regarding materials, methods, synthetic procedures, and spectral data can be found in the previously published paper^10^.

#### Apparatus, materials, and analysis

Dichloromethane, methanol, and dimethylformamide were supplied from Sigma Aldrich (Saint Louis, MO, USA). Ciprofloxacin (98%) was purchased from Acros Organics (Geel, Belgium), and menthol (≥ 99%) and thymol (≥ 98.5%) were purchased from Sigma Aldrich (Saint Louis, MO, USA). All other chemicals were of analytical grade and were used without any further purification. The NMR spectra were recorded on a Bruker (Karlsruhe, Germany) AVANCE spectrometer (Bruker, Karlsruhe, Germany) operating at 300 MHz or 500 MHz for ^1^H NMR and at 75 MHz, or 125 MHz for ^13^C NMR. The spectra were measured in CDCl_3_ or CDCl_3_\CD_3_OD, 9:1 mixture, and are given as δ values (in ppm) relative to TMS. Mass spectral ESI measurements were carried out on LCT Micromass TOF HiRes apparatus (Micromass UK Limited, Manchester, UK). Melting points were determined on a Melting Point Meter KSP1D (A. Krüss Optronic, Hamburg, Germany) and were uncorrected. TLC analyses were performed on silica gel plates (Merck Kiesegel GF_254_, Merck, Darmstadt, Germany) and visualized using UV light or iodine vapor. Column chromatography was carried out at atmospheric pressure using Silica Gel 60 (230–400 mesh, Merck, Darmstadt, Germany) and using dichloromethane/methanol (0–6%) mixture as eluent.

### In vitro* tuberculostatic activity*

The synthesized compounds were examined in vitro for their tuberculostatic activity using the broth microdilution method according to CLSI M24, 3rd ed.^21^. Investigations were performed in 96-well microtiter plates by the twofold serial microdilution using Middlebrook 7H9 Broth medium (Beckton Dickinson) containing 10% of OADC (Beckton Dickinson). The inoculum was prepared from fresh LJ culture in Middlebrook 7H9 Broth medium with OADC, adjusted to a no. 0.5 McFarland tube, and diluted 1:100. The stock solution of the tested agent was prepared in DMSO. Each test compound stock solutions were diluted in Middlebrook 7H9 Broth medium with OADC by four-fold the final highest concentration to be tested. Compounds were diluted serially in sterile 96-well microtiter plates using 100 μl Middlebrook 7H9 Broth medium with OADC. Concentrations of tested agents ranged from 512 to 0.0625 µg/ml.

A growth control containing no antibiotic and a sterile control without inoculation were also prepared on each plate. The plates were incubated at 37 °C for 21 days. After the incubation period, 30 μl of Alamar blue solution was added to each well, and the plate was re-incubated for 24 h. Growth is indicated by a color change from blue to pink and the lowest concentration of compound that prevented the color change was noted as its MIC^22,23^. Isoniazid (INH) and Linezolid (LZD) as reference drugs were used for comparison.

### Synergism—antagonism evaluation

The combinations of tested derivative and reference drug were prepared in Middlebrook 7H9 medium (Becton–Dickinson, USA) containing 10% of OADC (Becton–Dickinson, USA). Each of the derivatives in concentrations ranging from 1 to 1/32 MIC was mixed with a reference drug in a concentration equal to 1/2 MIC. Simultaneously, each of the reference drugs (Isoniazid, Rifampicin, Ethambutol, and Streptomycin) in concentrations ranging from 1 to 1/32MIC was mixed with tested derivative in a concentration equal to 1/2MIC (MIC values of tested derivatives and reference drugs alone were assigned as described above). Then, all samples (4 ml) were supplemented with an inoculum of tested strains (1°McFarland; 12 μl) and incubated for 14 days at 37 °C. MIC of tested derivative in combination with a fixed concentration of reference drug (MICD/comb with RD) was defined as its lowest concentration of tested derivative combined with reference drug which inhibited the growth of microorganisms in a liquid medium. Similarly, MIC of reference drug in combination with a fixed concentration of tested derivative (MICRD/comb with D) was defined as its lowest concentration of reference drug combined with tested derivative which inhibited the growth of microorganisms in a liquid medium. FICI value was calculated according to the equation:

$$ {\text{FICI}} = \left( {{\text{MIC}}_{{{\text{D}}/{\text{comb}}.{\text{ with RD}}}} /{\text{MIC}}_{{\text{D alone}}} } \right) + \left( {{\text{MIC}}_{{{\text{RD}}/{\text{comb}}.{\text{ with D}}}} /{\text{MIC}}_{{\text{RD alone}}} } \right) $$

The obtained FICI values were then used to determine whether synergism (FICI ≤ 0.5), indifference (> 0.5 FICI ≤ 4), or antagonism (FICI > 4) occurred between the tested agents^24^.

### Lipophilicity and affinity to phospholipids

For HPLC analyses Prominence-1 LC-2030C 3D HPLC system (Shimadzu, Japan) equipped with a DAD detector and controlled by LabSolution system (version 5.90 Shimadzu, Japan) was used. Two different chromatography columns packed with surface-modified silica were utilized in this study:

1. Octyldecylsilane (C_18_ Hypersil GOLD™; 50 mm × 4.6 mm; 5.0 µm; Thermo Scientific, USA)
2. Immobilized artificial membrane (IAM.PC.DD2; 10 × 4.6 mm × 10.0 µm; Regis Technologies; USA).

Both columns were equipped with guard precolumn containing the same silica-modified bed as the main column. All measurements were carried out using the protocol proposed by Valko^19^ and co-workers and adopted in our laboratory. Briefly, methods developed by Valko offered the determination of Chromatographic Hydrophobicity Index (CHI) indices using one gradient elution experiment. Ammonium acetate buffer (50 mM, pH 7.4) and acetonitrile (ACN) were used as the mobile phase at a 1.5 mL/min flow rate. The gradient range varies depending on the column type:

1. For C_18_ starting from 2 to 98% ACN in 5.25 min. The maximum concentration of ACN was applied for 1.75 min.
2. For IAM starting from 0 to 85% ACN in 5.25 min. The maximum concentration of ACN was used for 0.5 min.

During the chromatographic separation, the temperature was stable at 30 °C. Retention times (tR) at wavelength 280 nm were collected for all investigated Ciprofloxacin derivatives. The injected volume was 10 μL, whereas the concentration of solutes was at level 100 μg/mL. All studied compounds were dissolved in DMSO. Each chromatographic experiments run in triplicate. The CHI_C18_ and CHI_IAM_ indices of target Ciprofloxacin derivatives were obtained using the reference substances. Retention times for studied molecules and reference standards are collected in supplementary materials, Tables S1, S2 and S3, respectively.

Next, the CHI_C18_ values were converted into lipophilicity indices CHI log D using Eq. (1).

1 $$ {\text{CHI logD}} = 0.0{525}*{\text{CHI}}_{{{\text{C18}}}} {-}{1}.{467} $$

Analogical log *k*_IAM has_ been calculated using Eq. (2):

2 $$ {\text{Log}}k_{{{\text{IAM}}}} = 0.0{45}*{\text{ CHI}}_{{{\text{IAM}}}} + 0.{42} $$

The received CHI log D and log *k*_IAM_ were collected in Table 4.

### Quantitative structure-retention relationship

The quantitative structure-retention relationship (QSRR) approach was applied to evaluate the influences of molecular structures to determine lipophilicity and phospholipophilicity. Theoretical descriptors, which quantitatively describe target Ciprofloxacin derivatives, were calculated using chemicalize software (https://chemicalize.com), and are listed in supplementary materials, Table S4, except for the logD value of pH 7.4 given in Table 1, together with the experimentally determined lipophilicity.

QSRR models were calculated using multiple linear regression (MLR) algorithms where log *k*_IAM_ or CHI logD were dependent variables and theoretical descriptors independent. Before QSRR analysis, all data were preprocessed using feature standardization. The selection of descriptors was supported by a genetic algorithm (GA) mode applying a self-programmed Python script, where population size, number of algorithm iterations, and the maximum number of algorithm re-tries were 500, 200, and 200, respectively. Before the models’ calculation, Ciprofloxacin derivatives were split into training and validation sets using a 1:4 ratio. The model fitting, robustness, and predictive abilities were assessed by the coefficient of determination (R^2^), correlation coefficient of leave-one-out validation (Q^2^_LOO_), predictive squared correlation coefficient (Q^2^), root-mean-squared error of cross-validation (RMSE_CV_) and root mean-square error in prediction (RMSE_P_).

### Molecular docking

Three models of DNA gyrase were created using the crystal structure of topoisomerase II (DNA gyrase) complexed with DNA (PDB ID: 5BTC^16^) and the information about mutations associated with fluoroquinolone resistance^25^. The first model involved four-point mutations (D89G, S90V, S91P, and D94A) in the A subunit (GYR A) region, while the second model incorporated four-point mutations (R482G, N499G, T500H, and E501G) in the B subunit (GYR B) region. The third model combined all eight-point mutations in both the A and B subunits. The models were constructed using Modeller version 10.4^26^. Ligand structures generated in our previous work^10^ were used to perform simulations using AutoDock4 (v. 4.2) and AutoDockTools4^27^, and a similar docking procedure was carried out. For each ligand involved 1000 independent docking simulations were performed using a genetic algorithm with local search (GA-LS). This resulted in 1000 conformers with the lowest binding energy. The resulting structures were clustered with an RMSD cutoff at 3 Å to identify the most preferred binding modes. The final ligand-docked poses were selected as the central structures of the largest identified clusters.

### X-ray data

For this work, we used X-ray data related to single crystal structures and the crystal structure of topoisomerase II (DNA gyrase) complexed with DNA (PDB ID: 5BTC). X-ray structures were chosen from Cambridge Structural Database with the following REFCODEs:

- CIPROFLOXACIN—UHITOV01^28^
- MOXIFLOXACIN—ABABIQ (chloride monohydrate)^29^
- GATIFLOXACIN—HOTTOA (hydrochloride)^30^
- LEVOFLOXACIN—LICWOM (monoclinic), LICWOM01 (orthorhombic)^31^

Ciprofloxacin complex with DNA gyrase was the same as used in molecular docking experiment^16^.

### Supplementary Information

Supplementary Information.

## Data Availability

The datasets generated and analyzed during the current study are available from the corresponding author upon reasonable request.
