# Supplementary material for: Menthol- and thymol-based ciprofloxacin derivatives against Mycobacterium tuberculosis: in vitro activity, lipophilicity, and computational studies
Source: Sci Rep. 2023 Sep 28;13:16328. doi: 10.1038/s41598-023-43708-4 (PMC10539350; doi:10.1038/s41598-023-43708-4)

Supplementary file

Menthol- and thymol-based ciprofloxacin derivatives against *Mycobacterium tuberculosis*: *in vitro* activity, lipophilicity, and computational studies.

Daniel Szulczyk[a*], Mateusz Woziński[b], Michał Koliński[c], Sebastian Kmiecik[d], Agnieszka Głogowska[e], Ewa Augustynowicz-Kopeć[e], Michał A. Dobrowolski[f], Piotr Roszkowski[f], Marta Struga[a], Krzesimir Ciura[b,g].

[a] *Chair and Department of Biochemistry, Medical University of Warsaw, 02-097 Warsaw, Poland*

[b] *Department of Physical Chemistry, Medical University of Gdańsk, 80-416 Gdańsk, Poland*

[c] *Bioinformatics Laboratory, Mossakowski Medical Research Institute, Polish Academy of Sciences, 5 Pawinskiego St., 02-106 Warsaw, Poland*

[d] *Biological and Chemical Research Centre, Faculty of Chemistry, University of Warsaw, 02-089 Warsaw, Poland*

[e] *Department of Microbiology, National Tuberculosis and Lung Diseases Research Institute, 01-138, Warszawa, Poland*

[f] *Faculty of Chemistry, University of Warsaw, Pasteura 1, 02-093 Warszawa, Poland*

[g] *QSAR Lab Ltd., Trzy Lipy 3 St., 80-172 Gdańsk, Poland*

S1 *Quantitative structure-retention relationship*

Table S1. Retention times for all tested Ciprofloxacin derivatives achieved during biomimetic chromatography study.

Compound	IAM-HPLC					RP-HPLC				
**No.**	**tr_1**	**tr_2**	**tr_3**	**TM**	**SD**	**tr_1**	**tr_2**	**tr_3**	**TM**	**SD**
**1**	5.168	5.234	5.289	5.230	0.06	5.400	5.395	5.404	5.400	0.00
**2**	5.290	5.325	5.361	5.325	0.04	5.922	5.919	5.933	5.925	0.01
**3**	5.458	5.520	5.568	5.515	0.06	6.013	6.005	6.021	6.013	0.01
**4**	5.783	5.780	5.782	5.782	0.00	6.097	6.094	6.104	6.098	0.01
**5**	5.417	5.440	5.558	5.472	0.08	6.183	6.178	6.181	6.181	0.00
**6**	5.609	5.651	5.694	5.651	0.04	6.343	6.341	6.340	6.341	0.00
**7**	5.936	5.936	5.947	5.940	0.01	7.409	7.435	7.399	7.414	0.02
**8**	6.108	6.101	6.103	6.104	0.00	7.728	7.735	7.725	7.729	0.01
**9**	5.096	5.095	5.083	5.091	0.01	5.995	5.996	5.989	5.993	0.00
**10**	5.049	5.034	5.040	5.041	0.01	5.366	5.364	5.368	5.366	0.00
**11**	5.098	5.149	5.210	5.152	0.06	5.468	5.450	5.459	5.459	0.01
**12**	5.231	5.240	5.265	5.245	0.02	5.518	5.509	5.520	5.516	0.01
**13**	5.330	5.331	5.331	5.331	0.00	5.577	5.573	5.571	5.574	0.00
**14**	5.503	5.571	5.627	5.567	0.06	5.713	5.727	5.716	5.719	0.01

**15**	5.232	5.232	5.226	5.230	0.00	6.224	6.217	6.220	6.220	0.00
**16**	5.493	5.499	5.506	5.499	0.01	6.520	6.531	6.526	6.526	0.01

Table S2. Calibration set of compounds used on the C_{18} column together with their obtained retention data and literature CHI index.

**Compound**	**tr_1**	**tr_2**	**tr_3**	**t_{mean}**	**SD**	**CHI_{C18}**
Theophylline	2.172	2.180	2.175	2.176	0.004	18,4
Benzimidazole	2.599	2.610	2.603	2.604	0.006	34,3
Colchicine	3.265	3.275	3.267	3.269	0.005	42.0
Acetophenone	3.572	3.582	3.573	3.576	0.006	65,1
Indole	3.981	3.993	3.987	3.987	0.006	71,5
Propiophenone	4.120	4.131	4.122	4.124	0.006	77,5
Butyrophenone	4.534	4.545	4.536	4.538	0.006	87,5
Valerophenone	4.897	4.907	4.899	4.901	0.005	96,2

t- retention time, t_{mean} – mean value of retention time, CHI_{C18} - chromatographic hydrophobicity indexes

Table S3. Calibration set of compounds used on the IAM column together with their retention data and CHI index.

**Compound**	**tr_1**	**tr_2**	**tr_3**	**t_{mean}**	**SD**	**CHI_{IAM}**
Paracetamol	2.421	2.381	2.383	2.395	0.02	2.9
Acetanilidine	2.718	2.689	2.692	2.700	0.02	11.5
Acetophenone	3.292	3.277	3.281	3.283	0.01	17.2
Propiophenone	3.739	3.727	3.732	3.733	0.01	25.9
Butyrophenone	4.117	4.106	4.111	4.111	0.01	32
Valerophenone	4.696	4.683	4.689	4.689	0.01	37.3
Hexanophenone	4.927	4.913	4.919	4.920	0.01	41.8
Heptanophenone	5.214	5.202	5.208	5.208	0.01	45.7
Octanophenone	5.660	5.648	5.655	5.654	0.01	49.4

t- retention time, t_{mean} – mean value of retention time, CHI_{IAM} - chromatographic hydrophobicity indexes of immobilized artificial membrane chromatography

Table S4. Theoretical descriptors calculated by chemicalize software for investigated ciprofloxacin derivatives.

No.	Van der Waals volume	Van der Waals surface area	Solvent accessible surface area	Topological polar surface area	Minimum projection area	Maximum projection area	Minimum projection radius	Maximum projection radius	Charge pH 7.4	log D pH 7.4	Strongest acidic pKa	Strongest basic pKa	Atom count	Heavy atom count	Asymmetric atom count	Rotatable bond count	Ring count	Aromatic ring count	Hetero ring count	FSP3	Hydrogen bond donor count	Hydrogen bond acceptor count	Formal charge	Topological polar surface area	Polarizability	Molar refractivity
1	485.12	782.01	824.04	90.39	67.5	146.15	5.66	10.91	0.0165	2.4872	5,33	12,19	73	39	0	9	5	3	2	0.43	1	7	0	90.39	55.3	147.8
2	506.53	820.99	809.72	90.39	63.53	152.81	5.58	11.7	0.0165	2.2536	5,33	12,19	79	39	3	9	5	2	2	0.63	1	7	0	90.39	56.21	147.97
3	523.01	852.71	831.44	90.39	79.98	152.27	6.41	11.08	-0.0958	2.6946	5,33	8,3	82	40	3	10	5	2	2	0.65	1	7	0	90.39	58.04	152.73
4	502.2	812.1	877.96	90.39	77.96	152.04	6.01	9.46	-0.0958	2.9281	5,33	8,3	76	40	0	10	5	3	2	0.45	1	7	0	90.39	57.13	152.55
5	720.14	1184.45	1264.83	105.69	105.97	204.6	7.48	12.39	0.8865	8.511	8,29	-	110	56	0	18	6	4	2	0.47	0	7	0	105.69	82.28	216.64
6	541.22	882.26	877.49	90.39	74.16	164.35	6.23	12.9	-0.011	3.0293	5,33	8,95	85	41	3	11	5	2	2	0.66	1	7	0	90.39	59.88	157.33
7	520.51	843.22	883.12	90.39	74.6	156.37	5.97	11.8	-0.011	3.2629	5,33	8,95	79	41	0	11	5	3	2	0.47	1	7	0	90.39	58.97	157.15
8	794.56	1332.94	1294.19	105.69	124.57	220.03	8.07	14.84	0.9722	8.3259	-	8,94	128	58	6	20	6	2	2	0.74	0	7	0	105.69	87.77	226.19
9	555.89	914.49	902.31	90.39	75.22	163.75	5.75	12.33	-0.0109	3.4739	5,33	8,95	88	42	3	12	5	2	2	0.67	1	7	0	90.39	61.71	161.93
10	811.65	1352.00	1258.11	79.39	105.57	233.25	7.95	17.34	0.9722	10.0525	-	8,94	132	58	6	20	6	2	2	0.73	0	6	0	79.39	90.42	233.5
11	535.43	871.68	932.97	90.39	80.79	165.58	6.35	10.77	-0.0109	3.7074	5,33	8,95	82	42	0	12	5	3	2	0.48	1	7	0	90.39	60.8	161.75
12	788.17	1306.05	1379.37	105.69	117.1	226.41	8.36	14.29	0.9722	9.6815	-	8,94	122	60	0	22	6	4	2	0.51	0	7	0	105.69	89.63	235.04
13	488.63	790.15	778.16	90.39	68.43	154.53	6.28	11.47	-0.9833	2.4104	5,63	-1,41	76	38	3	7	5	2	2	0.62	1	7	0	90.39	54	143.14
14	489.5	795.07	783.73	90.39	67.83	151.06	5.67	11.15	-0.9475	2.9712	5,41	6,04	76	38	3	8	5	2	2	0.62	1	7	0	90.39	54.37	143.27
15	467.19	751.3	808.09	90.39	72.09	136.97	5.4	9.85	-0.9524	3.2064	5,38	6	70	38	0	8	5	3	2	0.41	1	7	0	90.39	53.46	143.1
16	466.82	753.43	795.12	90.39	65.69	141.88	5.95	11.25	-0.9833	2.677	5,63	-1,44	70	38	0	7	5	3	2	0.41	1	7	0	90.39	53.09	142.7
17	282.81	440.5	476.26	72.88	43.25	97.73	5.02	7.53	-0.0247	-0.8859	5,33	8,77	42	24	0	3	4	2	2	0.41	2	6	0	72.88	32.27	87.94

Figure 1S. Williams plots of obtained models, results of y-randomization test, and observed vs. predicted for IAM (A) and C_{18} bonded stationary phase (B)

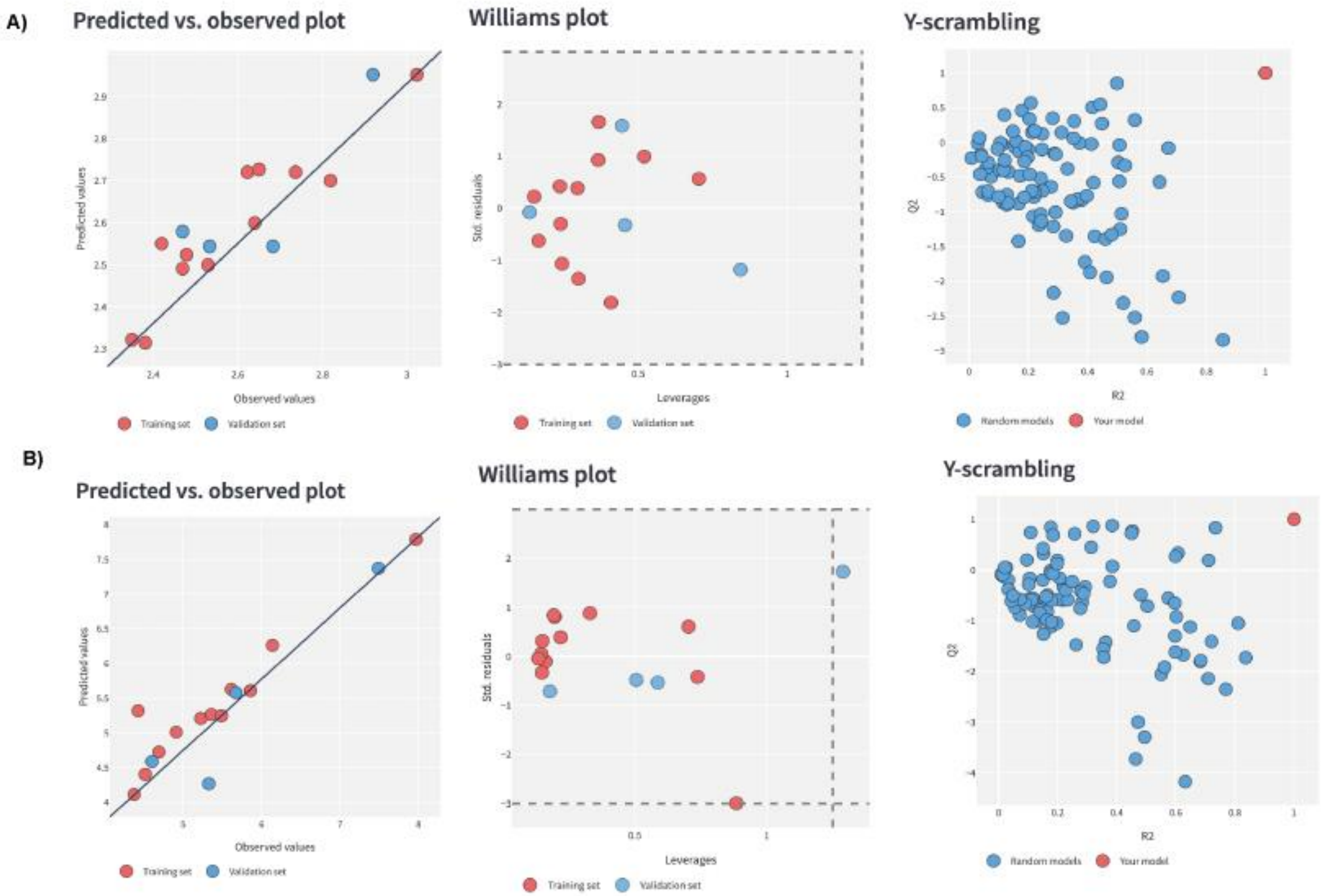

Supplement: Supplementary file 1 — Supplementary Information. [file 41598_2023_43708_MOESM1_ESM.pdf]
